# Quality of Life in Children Diagnosed With Non-classic Congenital Adrenal Hyperplasia

**DOI:** 10.7759/cureus.18937

**Published:** 2021-10-21

**Authors:** Maria João Ferreira, Rita Moita, Marta Canha, Sofia Ferreira, Carla Costa, Paulo Almeida, José Luís Castedo, Davide Carvalho, Cintia Castro-Correia

**Affiliations:** 1 Endocrinology, Diabetes and Metabolism, Sao Joao Hospital Center, Porto, PRT; 2 Pediatrics, Sao Joao Hospital Center, Porto, PRT; 3 Psychology, Sao Joao Hospital Center, Porto, PRT

**Keywords:** children quality of life, health related quality of life, non classical congenital adrenal hyperplasia, congenital adrenal hyperplasia, quality of life

## Abstract

Background

Non-classical congenital adrenal hyperplasia (NC-CAH) is a chronic disease characterised by^ ^excessive androgen production that may negatively affect the quality of life (QoL) of affected patients. Pediatric Quality of Life Inventory 4.0 (PedsQL™) is a validated tool to assess health-related QoL (HRQoL).

Methods

A cross-sectional study including 19 patients with NC-CAH was carried out in the pediatric endocrinology department. NC-CAH patients who agreed to participate were included. Anthropometric data* *was collected. PedsQL™ was applied to the patients and their parents. Patients were divided into four groups according to age: 2-4, 5-7, 8-12, and 13-18 years old. The control group consisted of healthy individuals from the instrument's validation studies for the Portuguese population and the standard control population used in the PedsQL™ validation study.

Results

The only difference found concerns the parents' score results for children aged 8-12, which showed physical health and emotional dimension scores significantly higher (86.16±9.86 vs.68.90±23.02 p=0.004, 69.17±14.14 vs. 65.82±19.24 p=0.004), while psychosocial health's score and total scale score were significantly lower than the control group (59.99±9.90 vs. 69.34±14.07 p=0.047, 73.11±4.65 vs.78.86±16.61 p=0.017).

Conclusion

HRQoL scores are not negatively affected by NC-CAH in most group ages, with the exception of the parents' reports on HRQoL for children aged 8-12. Further studies with a greater number of patients are needed to determine the impact of this chronic disease on the HRQoL of children.

## Introduction

Congenital adrenal hyperplasia (CAH) is an autosomal recessive disorder of the cortisol synthesis pathway that is most commonly caused by the mutation of the *CYP21A1* gene [1,2]. This results in an enzyme deficiency in the adrenal cortex leading to, in over 90% of the cases, 21-hydroxylase deficiency (21-OHD) [1,3-6]. The hallmark of this disease is excessive androgen production, resulting from the impaired or no conversion of 17-hydroxy-progesterone (17-OHP) to 11-deoxycortisol and of progesterone to deoxycorticosterone [7]. The blockade of steroid conversion results in increased androgen precursors production under corticotropin-releasing hormone-adrenocorticotropic hormone (CRH-ACTH) stimulation, leading to biochemical hyperandrogenism, marked by elevated 17-OHP levels, with variable gravity according to different phenotypes [1,2,4,6,8,9]. As so, the disease presents a broad spectrum, ranging from severe forms (classical CAH) to mild forms (non-classical CAH). The non-classical form (NC-CAH) is characterised by a less severe deficiency and manifests as increasing virilisation before puberty without genital ambiguity [2,10]. NC-CAH is more prevalent than the classic form, estimated to affect 0.1-0.2% of the population [11]. It is also more frequently observed in females, as males with NC-CAH have less recognized signs of androgen excess [12]. Androgen excess in NC-CAH patients can lead to premature pubarche in children and ovarian hyperandrogenism in adolescence. Affected patients may present signs of androgen excess, such as acne, hirsutism, apocrine body odour, irregular menstruation, and advanced bone age, which may lead to short adult height [2,13].⁠

In contrast to classical CAH patients, adrenal replacement is not required in NC-CAH patients [14]. Pharmacological treatment is focused on the management of signs of hyperandrogenism [12]. Children with NC-CAH should be treated for inappropriately early onset of body hair and odour when bone maturation is sufficiently accelerated to adversely affect future height. Clinicians can withhold treatment with careful monitoring if advanced bone age is not present [14]. In children, the treatment consists of glucocorticoids (GC) in doses aimed at suppressing hyperandrogenism, which are usually higher than the physiologic replacement and do not mimic the physiological circadian rhythm of cortisol [13]. In adolescents with irregular menstruation and acne, symptoms are usually reversed within three months of treatment with GC, whereas hirsutism remission is more difficult with GC monotherapy. In such cases, an oral contraceptive with or without anti-androgens is likely the best approach. Once near-adult height has been reached, tapering and discontinuing GC treatment should be considered [14].

Awareness of a medical condition, symptoms of hyperandrogenism and consequent virilisation, the burden of daily medication administration, the fear of an adrenal crisis, and fluctuating adrenal androgen levels may negatively impact the quality of life (QoL) of CAH patients [10,11,15].

Health-Related QoL (HRQoL) has been increasingly used as a supplement to clinical treatments, ensuring that the patients' perspective on the disease, the need for care, and their preferences for treatment modalities are considered. HRQoL in patients with CAH (including NC-CAH) has been widely studied; however, to our knowledge, investigation on HRQoL of NC-CAH patients exclusively is scarce [11,16].

We aimed at evaluating the health-related quality of life of pediatric patients with NC-CAH. We report scores from the validated Pediatric Quality of Life Inventory 4.0 (PedsQL™) [17].

## Materials and methods

A cross-sectional study including 19 patients with NC-CAH was carried out in our pediatric endocrinology department. Patients diagnosed with NC-CAH who agreed to participate were included. NC-CAH diagnosis was made by elevated 17-OHP levels in the cosyntropin stimulation test, which is accepted as the gold standard for NC-CAH diagnosis [14]. All patients were referred to genetic consultation, and all had *CYP21A2* gene mutations. Exclusion criteria were refusal and/or incapacity to answer the questionnaire and the presence of other comorbidities which may interfere with results. No patient or parent refused to participate. Three patients were excluded as they missed the medical appointment and the medical staff could not contact them. One child was excluded because of the presence of multiple comorbidities not related to the disease, which could possibly affect the assessment of HRQoL.

Clinical follow-up of patients in our unit included measurements of weight (by a standard calibrated scale) and height (by a stadiometer) of patients. Body mass index (BMI) was calculated as weight in kilograms divided by height in meters squared, and the growth chart percentiles of the Centers for Disease Control and Prevention were registered.

Patients were divided into four different groups according to their ages: 'toddlers' - 2-4 years old (two patients); 'young children' - 5-7 years old (one patient); 'children' - 8-12 years old (seven patients) and 'adolescents' - 13-18 years old (nine patients). The Portuguese version of Pediatric Quality of Life Inventory 4.0 (PedsQL™) was used to evaluate HRQoL. PedsQL™ has been widely used in previous studies that assessed HRQoL in children and adolescents. It has been translated and validated for use in the Portuguese population [17,18]. It presents different versions for different age groups, respecting the cognitive development of children. For children, the following versions are available: 5-7 years (interview), 8-12 years, and 13-18 years of age. For parents, versions of 2-4, 5-7, 8-12, and 13-18 years age groups are available. Its questions cover physical functioning (eight items), emotional dimension (five items), social dimension (five items), and scholar dimension (five items). The items of the different age versions are essentially similar, differing only in the appropriate language for each stage of cognitive development. Items were scored on a 5-point Likert scale for children, adolescents, and parents' versions. On this scale, zero (0) means never, and four (4) means almost always. In the 5-7 years old children versions, the faces scale uses the anchors 0 (never), 2 (often), and 4 (very often). Items are inversely scored and linearly transformed from zero (0) to one hundred (100) (0 = 100, 1 = 75, 2 = 50, 3 = 25 and 4 = 0). Scores were obtained for each of the measuring scales and grouped into two major dimensions: physical health (physical functioning items), psychosocial health (emotional, social, and school functioning items). A total score was also obtained by dividing the sum of all items by the number of the items with a valid answer. Higher scores point to a better HRQoL. If more than 50% of items are missing for a scale, the score was not given.

The group used as control consisted of healthy individuals from the instruments' previously published validation studies for the Portuguese population of children between age 5-7 years (which included 68 'young children') and 8-12 years (which included 111 'children') [17,18]. For patients aged 2-4 and 13-18 years, the results of the study survey were compared to a standard control population used in the PedsQoL validation study by Varni et al. that included 3070 toddlers and 1170 adolescents [19]⁠⁠.

All the categorical variables were described using frequencies and percentages, and the continuous variables were presented using means and standard deviations or median and minimum and maximum value, as appropriate. One sample T-test was performed for associations between PedsQL scores by the five groups and those reported by groups used as controls. Children's and parents' reports were compared using the Mann-Whitney-U test. Statistical analysis was not performed for the 5-7 years age group as there was only one patient in this age group. A p-value of <0.05 was considered significant. Statistical analysis was performed with the Statistical Package for the Social Sciences (SPSS) software, version 20.0 (IBM, Armonk, NY). This study was approved by the Hospital Ethical Committee (Project number 419-20, Date 17/12/2020). All eligible participants agreed to participate in the study and provided parental informed consent.

## Results

Characterization of the population

Nineteen patients, 13 (68.4%) girls and six (31.6%) boys, were included in the study. The median age was 11.9±4.9 years. Regarding diagnosis, seven (36.8%) patients were referred to a pediatric endocrinologist for signs of premature pubarche, four (21.1%) for family history, three (15.8%) for axillary apocrine odour, three (15.8%) for hirsutism, one (5.3%) for oligomenorrhea and one (5.3%) were diagnosed while studying an adrenal mass. The mean BMI was 20.2 kg/m^2^, corresponding to a mean percentile (Pc) of 61.9. Five patients (26.3%) were overweight, and two (10.5%) were obese. Of those, three female (15.8%) and two (10.5%) male patients were overweight; two (10.5%) male patients were obese. Boys with excess weight corresponded to 66.7% of the male patients. Fifteen (84.2%) patients were under treatment with hydrocortisone. The characteristics of patients are summarized in Table 1. 

**Table 1:** Patients characteristics n = Number of patients; Other = Work-up of an adrenal mass

Characteristics	Female patients	Male patients	Total
Patients, n (%)	13 (68.4)	6 (31.6)	19 (100)
Age (median)	13.4	8.7	11.9±4.9
Signs/symptoms			
Premature adrenarche, n (%)	6 (46.2)	1 (16.7)	7 (36.8)
Axillary apocrine odour, n (%)	0 (0.0)	3 (50.0)	3 (15.8)
Hirsutism, n (%)	3 (23.1)	0 (0.0)	3 (15.8)
Oligoamenorhea, n (%)	1 (7.7)	-	1 (5.3)
Family history, n (%)	3 (23.1)	1 (16.7)	4 (21.1)
Other, n (%)	0 (0.0)	1 (16.7)	1 (5.3)
BMI (kg/m^2 ^mean, mean Pc)	20.0 (55.5)	20.6 (74.7)	20.2 (61.9)
Hydrocortisone treatment n (%)	12 (91.7)	4 (66.7)	16 (84.2)
Dose of hydrocortisone per body surface area (mean, mg/m^2^/day)	7.8	7.2	7.5

HRQoL assessments

HRQoL assessments are summarized in Tables 2-5. 

**Table 2:** PedsQL scores Min = minimum value; Max = maximum value Median, minimum and, maximum not computed for 5-7 age for lack of data (only one patient completed the questionnaire).

PedsQL scores		2-4 age group (*n*=2, 10.5%)	5-7 age group (*n*=1, 5.3%)	8-12 age group (*n*=7, 36.8%)	13-18 age group (*n*=9, 47.4%)
		median (min-max)	median (min-max)	median (min-max)	median (min-max)
Children’s reports	Total scale score	--	85.40	81.04 (68.33-93.30)	82.81 (69.30-99.20)
	Physical health	--	87.50	93.75 (65.00-100)	84.40 (63.50-100.00)
	Psychosocial health	--	83.30	71.7 0(65.00-86.70)	85.00 (66.70-98.30)
	Emotional functioning	--	80.00	72.50 (45.00-75.00)	75.00 (35.00-100.00)
	Social functioning	--	100.00	85.00 (65.00-100.00)	90.00 (75.00-100.00)
	School functioning	--	70.00	67.50 (55.00-85.00)	85.00 (70-95.00)
Parents' reports	Total scale score	75.20 (69.80-80.60)	76.50	74.20 (63.13-76.70)	85.85 (65.50-99.20)
	Physical health	81.63 (71.90-90.63)	81.30	81.25 (75.00-100.00)	90.63 (59.40-100.00)
	Psychosocial health	69.20 (70.60-67.80)	71.80	61.70 (45.00-73.30)	76.67 (98.30 - 68.30)
	Emotional functioning	80.00 (70.00-90.00)	50.00	45.00 (30.00-55.00)	55.00 (45.00-100.00)
	Social functioning	65.00 (55.00-75.00)	100.00	72.50 (35.00-100.00)	90.00 (75.00-100.00)
	School functioning	62.50 (58.30-66.70)	65.00	65.00 (45.00-70.00)	90.00 (75.00-95.00)

**Table 3:** Comparison between scores of 5-7 and 8-12 years children with the Portuguese control group s.d. = Standard deviation; * = statistically significant Standard deviation and p-value not computed for 5-7 years age group due to lack of data (only one patient completed the questionnaire).

PedsQL scores		5-7 age study group (n=1, 5.3%)	5-7 age control group	p-value	8-12 age study group (n=7, 36.8%)	8-12 age control group	p-value
		value	mean (± s.d.)		mean (± s.d.)	mean (± s.d.)	
Children's reports	Total scale score	85.40	75.45 (± 12.04)	--	80.38 (± 9.11)	75.74 (±14.27)	0.226
	Physical health	87.50	77.39 (18.±42)	--	87.41 (±13.31)	78.04 (±19.55)	0.110
	Psychosocial health	83.30	74.41 (±10.99)	--	73.34 8±6.95)	74.52 (±13.81)	0.675
	Emotional functioning	80.00	74.71 (±15.88)	--	65.83 (±12.81)	70.05 (±18.25)	0.452
	Social functioning	100.00	76.32 (±14.85)	--	85.00 (±14.14)	80.72 (±17.84)	0.489
	School functioning	70.00	72.21 (±17.69)	--	69.17 (±11.58)	72.79 (±15.16)	0.477
Parents' reports	Total scale score	76.50	68.78(±17.28)	--	73.11 (±4.65)	69.19 (±15.48)	0.068
	Physical health	81.30	68.40 (±21.40)	--	86.16 (±9.86)	68.90 (±23.02)	0.004*
	Psychosocial health	71.80	68.98 (±16.88)	--	59.99 (± 9.90)	69.34 (±14.07)	0.047*
	Emotional functioning	50.00	65.83 (±16.97)	--	69.17 (±14.14)	65.82 (±19.24)	0.004*
	Social functioning	100.00	75.42 (±21.92)	--	69.17 (±21.31)	76.23 (±20.01)	0.456
	School functioning	65.00	65.69 (±21.32)	--	61.67 (±8.76)	65.98 (±16.04)	0.289

**Table 4:** Comparison between the present study sample and original study sample by Varni et al. [19] s.d. = standard deviation; * = statistically significant Standard deviation and p-value for 5-7 years age group, and p-value for 2-4 years age group were not computed due to lack of data.

PedsQL scores		Study sample		Varni et al. sample		p-value
		mean	s.d.	mean	s.d.	
Children's reports	Total scale score 2-4 age group (n=2, 10.5%)	--	--	--	--	
	Total scale score 5-7 age group (n=1, 5.3%)	85.40	---	81.86	12.64	--
	Total scale score 8-12 age group (n=7, 36.8%)	80.38	9.11	83.31	13.45	0.427
	Total scale score 13-18 age group (n=9, 47.4%)	84.24	9.18	83.65	13.30	0.851
Parents' reports	Total scale score 2-4 age group (n=2, 10.5%)	75.20	5.40	87.42	12.49	--
	Total scale score 5-7 age group (n=1, 5.3%)	76.50	--	78.02	16.44	--
	Total scale score 8-12 age group (n=7, 36.8%)	73.11	4.65	78.86	16.61	0.017*
	Total scale score 13-18 age group (n=9, 47.4%)	86.67	9.01	79.45	16.40	0.264

**Table 5:** Comparison between children's and parents' reports.

PedsQL scores	Children's reports total scale score median (min-max)	Parents' reports total scale score median (min-max)	p-value
8-12 age group (n=7, 36.8%)	81.04 (68.33-93.30)	74.20 (63.13-76.70)	0.126
13-18 age group (n=9, 47.4%)	82.81 (69.30-99.20)	85.85 (65.50-99.20)	0.857
Total (n=16, 84.2%)	82.51 (68.33-99.20)	76.53 (66.13-99.20)	0.347

Toddlers (2-4 years)

Parents' HRQoL Scores

Parents' reports on the total score of the 2-4 years age group were similar to the one reported by Varni* *et al. (75.20 ± 5.40 vs. 87.42 ± 12.49). Statistical analysis was not computed due to the small sample size.

Young children (5-7 years)

Self-Assessment HRQoL Scores

This group consisted of only one patient. Even though it was not possible to perform statistical analysis compared to the control population, our patient presented a higher score in every item of the questionnaire. The child's self-assessment scores were similar to those reported by Varni et al. (85.40 vs. 81.86±12.64).

Parents' HRQoL Scores

HRQoL score assessed by the parents had a higher score than the mean of the Portuguese control group for every item, besides the parent's report on emotional functioning (50.00 vs. 68.83± 16.97) and school functioning (65.00 vs. 65.69 ± 21.32). 

Parents' report score on total scale score in the 5-7 years age group was similar to the one reported by Varni et al. (76.50 vs. 78.02±16.61). Statistical analysis was not performed due to the small sample size.

Children (8-12 years) 

Self-Assessment HRQoL Scores

HRQoL scores self-evaluated by children aged 8-12 years were similar to those obtained by the Portuguese control group. Total scale score, physical health, and social functioning scores were higher than the ones reported by the Portuguese control group (80.38±9.11 vs. 75.74±14.27 p=0.226; 87.41±13.31 vs. 78.04±19.55 p=0.110; 85.00±14.14 vs. 80.72±17.84 p=0.489, respectively). On the other hand, psychosocial health, emotional dimension, and school dimension scores were lower than the ones reported by the same control group (73.34±6.95 vs. 74.52± 13.81 p=0.675; 65.83±12.82 vs. 70.05±18.25 p=0.452; 69.17±11.58 vs. 72.79±15.16 p=0.477).

Children's self-assessment score on total scale score for the 8-12 years age group was similar to the one reported by Varni et al. (80.38±9.11 vs. 83.31±13.45 p=0.427).

Parent's HRQoL Scores

Parent's reports of total HRQoL scores of children aged 8-12 were similar to those reported by the control group (73.11±4.65 vs. 69.19±15.48 p=0.068), as well as the social and school functioning score (69.17±21.31 vs. 76.23±20.01 p=0.456, 61.67±8.76 vs. 65.98±16.04 p= 0.289). Physical health and emotional dimension' scores were significantly higher than the control group (86.16±9.86 vs. 68.90±23.02 p=0.004, 69.17±14.14 vs. 65.82±19.24 p=0.004). Psychosocial health's score, on the other hand, was significantly lower than the one presented by the control group (59.99±9.90 vs. 69.34±14.07 p=0.047).

Parent's report score on total scale score on the 8-12 age group was significantly lower compared to the control sample by Varni et al. (73.11±4.65 vs. 78.86±16.61 p=0.017).

Adolescents (13-18 years)

Self-Assessment HRQoL scores

Older (13-18 years) children's self-assessment score on the total scale was similar to the control group (84.24±9.18 vs. 83.65±13.30 p=0.851) by Varni et al.

Parents' HRQoL Scores

Parents' report on total scale score (86.67±9.01 vs. 79.45± 16.40 p=0.264) was similar to the control group studied by Varni et al.

Parents' and children's HRQoL total scores

Parent's and children's reports on total scale score did not differ (78.62±10.06 vs. 82.72± 8.79 p=0.193), neither in 'young children' (80.38±9.11 vs. 73.11±6.95 p=0.084) nor in the 'adolescents' group (83.74±9.68 vs. 85.85±11.5 p=0.837).

## Discussion

Our study evaluated the quality of life of children with NC-CAH. Self-assessment and parent's reports for 'toddlers', 'young children', and 'adolescents' on HRQoL showed no significant overall difference between patients with NC-CAH and control groups. Parents' report score results of children aged 5-7 years were lower on emotional and school functioning, however, without statistical significance. This is in line with previous reports of Brener et al., who examined NC-CAH patients and compared them to their healthy siblings and showed no difference between the total scores and the scale and sub-scale scores for HRQoL [11]. A Dutch study involving children with CAH, including NC-CAH, and their parents also showed no reduction in HRQoL of these patients [20].

Physical health scores of our study showed no differences between NC-CAH patients and control groups, except for parents' scores for children aged 8-12 years, which were significantly higher.

Psychosocial health scores, including emotional, social, and school dimensions, are similar between our study group and control groups in 'toddlers', 'young children' and 'adolescents'. This is in line with other studies that show that appropriately treated children and adolescents with NC-CAH have social and school dimension HRQoL scores similar to healthy populations [11]. This is encouraging as early exposure to hyperandrogenism may affect cognitive functions, including social behavior patterns, which is a major parental concern [11,21,22].

On the other hand, 'children' (aged 8-12 years) self-assessment HRQoL scores showed lower results on psychosocial health, emotional dimension, and school dimension scales compared to the control group, however, without statistical significance. Parents' HRQoL scores were significantly lower on the psychosocial health scale than the Portuguese control group, social and school dimensions being the items responsible for this result. The total scale score was also significantly lower in comparison to the control population studied by Varni et al. [19]. This has also been observed by Lima et al*.* [17] who demonstrated a lower total scale score by the Portuguese population compared to the control population by Varni et al. [19]. The authors hypothesized that this decline in quality of life may be associated with the phase of pre-adolescence, often characterised by changes in school and in personal relationships and new development tasks that lead to more responsibility for the child. Thus, this period may be associated with some socio-emotional instability that can explain this decline [17]. Brener et al. have also shown that the emotional score of NC-CAH patients and parents' scores were significantly lower compared to the healthy pediatric population of the USA [11]. Other studies have shown that parents of children with chronic disease over-report lower HRQoL compared to their children, including patients with CAH [15,23]. ⁠

Excess weight was present in 42.8% of our study. Interestingly, 50% of our patients with excess weight/obesity were between 8 and 12 years old, which was the age range that presented more differences with the control population. Obesity and increased metabolic risk have been reported in young patients with CAH and found to have a negative effect in many domains of HRQoL, namely on the physical, social, and school functioning domains [24,25]. Significantly impaired subjective health status in CAH adult patients (including NC-CAH patients), especially in the domains of general health, vitality, and role limitations due to emotional problems, as well as increased anxiety scores have also been demonstrated [26]. In our study, however, significantly higher results on physical and emotional functioning scores reported by the parents of children aged 8-12 years were observed. The fact that older children perceive themselves as more physically capable was also noted by Lima et al. [17]. Nonetheless, this is against what is usually observed in obese and overweight children [26].

Quality of life in adult patients with CAH (sometimes including NC-CAH patients) has been widely studied, some studies showing an overall impaired self-reported quality of life [27,28] while others showed no difference [26,29].

Study limitations 

Limitations to our study include the small sample size, which probably reflects the underdiagnosis and undertreatment of NC-CAH patients. Another limitation may be the lack of comparison between NC-CAH patients and similar-aged healthy children who share the same environment and the lack of a Portuguese control group for toddlers and adolescents. Our strengths include the uniformity of medical diagnosis (cosyntropin stimulation testing and genetic confirmation of *CYP21A2* gene mutations) and the adherence to treatment and surveillance in a single medical center.

## Conclusions

Our study examines the HRQoL in a pediatric cohort comprised solely of NC-CAH patients. Quality of life in adult patients with CAH has been extensively investigated, with conflicting results. Studies involving adolescents and children are limited, as well as those exclusively studying NC-CAH patients. Our findings suggest that HRQoL scores are not negatively affected by NC-CAH in most age groups, with the exception of the parents' reports on HRQoL of children aged 8-12 years. Parents' lower report on HRQoL has been previously described in children with chronic diseases. Our results are reassuring, as adequate treatment and follow-up of these patients seem to allow them to have a QoL similar to the healthy population. Nonetheless, attention should be directed to this particular group and understanding the reason for these lower scores. Further studies with a greater number of patients are needed in order to determine the impact of this chronic disease on the quality of life of children.

## References

[1] New MI (1992). Genetic disorders of adrenal hormone synthesis. Horm Res.

[2] Ghanny BA, Malhotra S, Kumta S, Kazachkova I, Homel P, Jacobson-Dickman E, Motaghedi R (2016). Should children with isolated premature adrenarche be routinely evaluated for non-classical congenital adrenal hyperplasia?. J Pediatr Endocrinol Metab.

[3] El-Maouche D, Arlt W, Merke DP (2017). Congenital adrenal hyperplasia. Lancet.

[4] Antal Z, Zhou P (2009). Congenital adrenal hyperplasia: diagnosis, evaluation, and management. Pediatr Rev.

[5] Riepe FG, Sippell WG (2007). Recent advances in diagnosis, treatment, and outcome of congenital adrenal hyperplasia due to 21-hydroxylase deficiency. Rev Endocr Metab Disord.

[6] Carvalho B, Marques CJ, Santos-Silva R, Fontoura M, Carvalho D, Carvalho F (2021). Congenital adrenal hyperplasia due to 21-hydroxylase deficiency: an update on genetic analysis of CYP21A2 gene. Exp Clin Endocrinol Diabetes.

[7] Livadas S, Bothou C (2019). Management of the female with non-classical congenital adrenal hyperplasia (NCCAH): a patient-oriented approach. Front Endocrinol (Lausanne).

[8] Carroll L, Graff C, Wicks M, Diaz Thomas A (2020). Living with an invisible illness: a qualitative study exploring the lived experiences of female children with congenital adrenal hyperplasia. Qual Life Res.

[9] Carvalho B, Pereira M, Marques CJ (2012). Comprehensive genetic analysis and structural characterization of CYP21A2 mutations in CAH patients. Exp Clin Endocrinol Diabetes.

[10] Zainuddin AA, Grover SR, Abdul Ghani NA (2020). Health-related quality of life of female patients with congenital adrenal hyperplasia in Malaysia. Health Qual Life Outcomes.

[11] Brener A, Segev-Becker A, Weintrob N (2019). Health-related quality of life in children and adolescents with nonclassic congenital adrenal hyperplasia. Endocr Pract.

[12] Macut D, Zdravković V, Bjekić-Macut J, Mastorakos G, Pignatelli D (2019). Metabolic perspectives for non-classical congenital adrenal hyperplasia with relation to the classical form of the disease. Front Endocrinol (Lausanne).

[13] de Vries L, Lebenthal Y, Phillip M, Shalitin S, Tenenbaum A, Bello R (2019). Obesity and cardiometabolic risk factors in children and young adults with non-classical 21-hydroxylase deficiency. Front Endocrinol (Lausanne).

[14] Speiser PW, Arlt W, Auchus RJ (2018). Congenital adrenal hyperplasia due to steroid 21-hydroxylase deficiency: an Endocrine Society Clinical Practice guideline. J Clin Endocrinol Metab.

[15] Halper A, Hooke MC, Gonzalez-Bolanos MT, Vanderburg N, Tran TN, Torkelson J, Sarafoglou K (2017). Health-related quality of life in children with congenital adrenal hyperplasia. Health Qual Life Outcomes.

[16] Daae E, Feragen KB, Nermoen I, Falhammar H (2018). Psychological adjustment, quality of life, and self-perceptions of reproductive health in males with congenital adrenal hyperplasia: a systematic review. Endocrine.

[17] Lima L, Guerra MP, Lemos MS (2009). Adaptation of the generic scale of the Pediatric Quality of Life Inventory — Pediatric Quality of Life Inventory 4.0 — PedsQL, to a Portuguese population (Article in Portuguese). Rev Port Saúde Pública.

[18] Ferreira PL, Baltazar CF, Cavalheiro L, Cabri J, Gonçalves RS (2014). Reliability and validity of PedsQL for Portuguese children aged 5-7 and 8-12 years. Health Qual Life Outcomes.

[19] Varni JW, Burwinkle TM, Seid M, Skarr D (2003). The PedsQL 4.0 as a pediatric population health measure: feasibility, reliability, and validity. Ambul Pediatr.

[20] Sanches SA, Wiegers TA, Otten BJ, Claahsen-van der Grinten HL (2012). Physical, social and societal functioning of children with congenital adrenal hyperplasia (CAH) and their parents, in a Dutch population. Int J Pediatr Endocrinol.

[21] Dorn LD, Hitt SF, Rotenstein D (1999). Biopsychological and cognitive differences in children with premature vs. on-time adrenarche. Arch Pediatr Adolesc Med.

[22] Karlsson L, Gezelius A, Nordenström A, Hirvikoski T, Lajic S (2017). Cognitive impairment in adolescents and adults with congenital adrenal hyperplasia. Clin Endocrinol (Oxf).

[23] Ingerski LM, Modi AC, Hood KK (2010). Health-related quality of life across pediatric chronic conditions. J Pediatr.

[24] Falhammar H, Frisén L, Hirschberg AL, Norrby C, Almqvist C, Nordenskjöld A, Nordenström A (2015). Increased cardiovascular and metabolic morbidity in patients with 21-hydroxylase deficiency: a Swedish population-based national cohort study. J Clin Endocrinol Metab.

[25] Pinhas-Hamiel O, Singer S, Pilpel N, Fradkin A, Modan D, Reichman B (2006). Health-related quality of life among children and adolescents: associations with obesity. Int J Obes (Lond).

[26] Arlt W, Willis DS, Wild SH (2010). Health status of adults with congenital adrenal hyperplasia: a cohort study of 203 patients. J Clin Endocrinol Metab.

[27] Johannsen TH, Ripa CP, Mortensen EL, Main KM (2006). Quality of life in 70 women with disorders of sex development. Eur J Endocrinol.

[28] Gilban DL, Alves Junior PA, Beserra IC (2014). Health related quality of life of children and adolescents with congenital adrenal hyperplasia in Brazil. Health Qual Life Outcomes.

[29] van der Kamp HJ, Slijper FM, Bullinger M (1996). The quality of life in adult female patients with congenital adrenal hyperplasia: a comprehensive study of the impact of genital malformations and chronic disease on female patients life. Eur J Pediatr.

